# Consumer acceptance of fungus-resistant grape wines: Evidence from Italy, the UK, and the USA

**DOI:** 10.1371/journal.pone.0267198

**Published:** 2022-04-27

**Authors:** Riccardo Vecchio, Eugenio Pomarici, Elisa Giampietri, Massimiliano Borrello

**Affiliations:** 1 Department of Agricultural Sciences, University of Naples Federico II, Naples, Italy; 2 Department of Land, Environment, Agriculture and Forestry, University of Padova, Padova, Italy; Groupe ESC Dijon Bourgogne, FRANCE

## Abstract

While there is evidence of consumers’ interest in wine sustainability, acceptance of innovations in wine production is not guaranteed. The current study addresses this issue by analyzing consumers’ acceptance of fungus-resistant grape (FRG) wines, a sustainable innovation that can substantially reduce the need for chemical inputs in viticulture. To do so, by means of an online survey including large samples of regular wine drinkers in Italy (N = 752), the UK (N = 858) and the USA (N = 856), the study compares individuals’ preferences for conventional wines with preferences for FRG wines. The study also explores whether FRG wine acceptance is influenced by informal or formal purchase occasion, by different types of information regarding the product, and by individual attitudinal characteristics. The findings show a general acceptance of FRG wines among consumers. In particular, consumers’ preferences for FRG wines on formal occasions are not significantly different from their preferences for conventional wine, whereas on informal occasions, consumers prefer FRG wines over conventional wines. Regarding the impact of information on participant choice, participants informed about the potential effects of FRG on sensory wine characteristics had lower preferences for FRG wines than those who read an information script regarding crop biodiversity. Last, individuals’ sustainability concerns and food technology neophobia had positive and negative influences on FRG acceptance, respectively. Overall, this research provides wineries, nurseries and policy-makers with important insights concerning the market potential of FRG wines in three key markets.

## 1. Introduction

Recently, sustainability concerns in winemaking have mirrored those of the agrifood sector at large [1], triggering several private and public initiatives. Starting in 2001 with the forerunner California Sustainable Winegrowing Alliance, environmental protection has been at the heart of international (*e*.*g*., the International Organization of Vine Wine ECO resolutions), national (*e*.*g*., sustainable winegrowing programs in many wine-producing countries, such as Australia, Chile, and Italy) and local (*e*.*g*., sustainability standards at the regional level in several European countries) actions. Moreover, the demand for more sustainable viticulture approaches has driven the uptake of organic [2], biodynamic [3], and natural [4] wines. This interest from the demand side translates into an increase in supply, as evidenced by the remarkable surge (+55%) in the EU-28 organic area under vine from 2013 (244 thousand hectares) to 2019 (379 thousand hectares) [5]. Sustainable viticulture approaches were mostly introduced to reduce the use of synthetic pesticides utilized by incumbent grapevine growers, especially against main cryptogamic diseases, such as downy and powdery mildews [6]. However, risks for plant and human health due to soil accumulation of sulfur- and copper-based products used in organic viticulture (*e*.*g*., the Bordeaux mixture) [7] have recently led the European Commission to impose restrictions on the use of copper in vineyards (Commission Implementing Regulation EU 2018/1981, [8]). Therefore, albeit these approaches are often considered a step forward in comparison to the conventional one, they still have controversial aspects, such that the international call for sustainable innovations in winemaking remains. Additionally, the reduced use of pesticides in the wine-making sector is of great interest to the public, representing an essential social demand [9].

In this context, fungus-resistant grape (FRG) varieties, also known as ‘PIWI’ (from German: Pilzwiderstandsf¨ahige, ‘disease resistant’), might be a gamechanger for the future of sustainable winemaking [10]. Resulting from the hybridization of the main Mediterranean grapevine (*i*.*e*., *Vitis vinifera*) with other *Vitis* species, FRG vines have innate resistance against cryptogamic diseases [11–14], so they rely less on chemical treatments. To illustrate, FRG wines allow a reduction in the use of synthetic pesticides by more than 80% [15], far greater than the 50% objective set by the European Green Deal. Some authors [16] argue that fungus-resistant grape varieties are also resistant to severe climate conditions. On the economic side, pesticides reduction can lead to approximately 58% savings/ha in the cost of treatments and 15% savings/ha in vineyard operating costs (data from the VINOVERT Interreg Sudoe European project, 2016–2019) [17]; while the transition to more environmentally sustainable production processes of operating with traditional varieties may have controversial effects on the economic sustainability of wine production [18,19].

Furthermore, as these novel genotypes derive from multiple ‘back-crosses’, they maintain a high percentage of the *V*. *vinifera* genome, thus preserving most of its sensorial properties [20]. Due to this information, considerable economic investments and research efforts have been recently devoted to developing and improving FRG wines. For example, a 25-year backcrossing breeding program in France has generated a set of genotypes in which disease resistance is conferred by one single gene for downy mildew and another single gene for powdery mildew [21]. Similarly, in Italy, some botrytis-tolerant cultivars have been produced through intraspecific controlled crosses [14]. A recent step forward is represented by resistance gene pyramiding [22,23], namely, a technique that allows the simultaneous control of several pathogens and combines different defense mechanisms to create durable multiple resistance traits.

For these reasons, FRG wines can play a pivotal role in the shift of winemaking toward environmentally sustainable production, generating economic gains for wine producers. However, while there is evidence of consumers’ interest in wine sustainability [1], individuals have complex preferences for wine characteristics [24], and their willingness to accept and purchase FRG wines cannot be taken for granted. Even though FRG varieties may include up to 99% of the original *V*. *vinifera* pedigree, FRG alter traditional wine production processes and may show a different sensory profile from that of wines produced with traditional grape varieties, thus affecting individuals’ acceptance of them. Furthermore, consumers might also be concerned that the uptake of resistant varieties will prevail over conventional vines, thus substantially altering local crop biodiversity. As the commercial exploitation of FRG wines is still in its infancy, producers require information concerning the consumer domain, which has not received much attention to date by researchers. Therefore, the main objective of the current research is to shed light on consumers’ acceptance of FRG wines. To do so, an experimental survey was designed and administered to large samples of regular wine consumers in three core markets, namely: Italy, the United Kingdom (UK) and the United States of America (USA). Consumers’ acceptance was assessed through a contingent valuation (CV) based on a linear payment ladder, eliciting participants’ maximum willingness to pay (WTP) for FRG wines in both a formal and informal purchase occasion. Additionally, respondents were randomly assigned to two different treatments to analyze the effect of diverse types of information on consumers’ preferences.

## 2. Research questions

Previous studies have shown that FRG sustainability characteristics positively affect some wine consumers [25–27]. However, these studies are limited in terms of representativeness of the global wine market. The current research aims to fill this void through a survey performed in three major countries for the global wine sector. Italy, the UK and the USA were selected to include Old World and New World relevant wine markets, also considering the national weight in terms of their relevance for wine production (Italy) and imports (the UK and the USA). Italy ranks first worldwide in wine production and exported volumes, second for the value of exportations and third in terms of overall wine consumption; the UK ranks globally first and second, respectively, in volume and value of imported wine; and the USA ranks first in wine consumption and total value of imported wine, and third in import volume [28].

Additionally, the current research draws from previous literature revealing that the purchasing occasion is an important determinant of consumer wine preferences. Individuals selecting the same product can behave differently depending on the occasion for which they are purchasing that good, particularly showing differences between purchases made for personal consumption versus other situations [29]. For wine, the literature has shown that wine purchase behavior is context specific [30]. More particularly, the social implications of formal occasions (*e*.*g*., a business dinner with colleagues or a gift) attribute a symbolic meaning to wine selection [31], leading individuals to increase their focus on quality characteristics in comparison to informal situations (*e*.*g*., a dinner at home with family or friends) [32].

Based on the previous considerations, our first and second research questions are the following:

RQ1) Do consumers in Italy, the UK and the USA accept FRG wines?
RQ2) Do Italian, British, and American consumers’ preferences for FRG wines vary according to informal or formal purchase occasions?

To answer these two questions, individuals’ maximum WTP for both conventional wine and FRG wine were collected through a linear payment ladder. The selection of this CV technique was due to its easiness to use and understand by participants; nevertheless, it should be noted that it is prone to issues of range and centering bias, potentially leading respondents to overestimate their WTP [33]. In the experimental survey individuals’ WTP was collected for FRG wines to be purchased for an informal occasion and a formal occasion. WTP distributions were analyzed via parametric and non-parametric tests.

Despite their sustainability characteristics, FRGs are a disruptive innovation in the wine sector that are tightly tied to tradition in terms of wine sensory properties and production methods [9]. At first, FRG hybrids generated very poor sensory wine characteristics due to an undesired ‘foxy’ off-flavor [11]. Although advanced backcrossing hybridization programs have solved this issue, FRG varieties are still likely to alter consumers’ perception of wine sensory profiles, particularly regarding wines selected for their peculiar sensory features. Furthermore, the opportunity to adopt FRG varieties has been questioned due to biodiversity concerns [10]. Reduced wine production costs due to FRG adoption can modify winemakers’ future choices. In the long run, this modification could lead to the full replacement of conventional grape varieties and, therefore, to the impoverishment of local crop biodiversity. In general terms, food technology neophobia, namely, consumers’ fear of novel food technologies [34], is a ‘fil rouge’ connecting potential threats to the commercialization of FRG wines [27]. There is wide evidence in the literature that consumers have often high perceived risk and aversion towards novel foods and associated food technologies [35]. As for the latter, increased consumers’ information on food processing frequently matches with a negative attitude regarding novel food technologies [36]. Even without evidence supporting wine sensory adulteration and long-term biodiversity impacts or other potential negative effects of FRG hybrids, individuals may be averse to the implementation of novel technologies in the wine sector, manifesting an *a priori* preference for traditional production methods. To consider these aspects and explore major criticalities of consumers’ acceptance of FRG wines, the current study includes in its design the investigation of consumers’ preferences under different information scenarios. Furthermore, it investigates whether individuals’ psychographic and socio-demographic characteristics potentially affect FRG acceptance. Considering this, our third and fourth research questions are the following:

RQ3) Do different types of information influence Italian, British, and American consumers’ preferences for FRG wines?
RQ4) What are the individual characteristics that impact Italian, British and American consumers’ preferences for FRG wines?

To respond to RQ3 the effect of information was assessed through a second round of WTP elicitation. Specifically, before this second round, the sample was randomly assigned to two different information scripts (half sample each), concerning two major criticisms of FRG adoption, that are the difference from the sensory profile of conventional counterparts and the potential impoverishment of crop biodiversity. The use of a split sampling lies in the desire to reflect a more realistic choice situation for the consumer, which is likely to be characterized by partial rather than complete information available to the consumer. Indeed, this is even more true for a radical innovation as FRG wines, for which the information available to the consumer is likely to be scarce.

Considering the panel structure of the collected WTP and its left and right censoring, a random effect tobit model was estimated to depict the impact of the type of information on individual valuations for formal and informal FRG wines. To answer RQ4, we collected three specific psychometric scales, to generate insights on participants’ wine involvement, food technology neophobia, and sustainability concerns. The effect of these variables and of socio-demographic characteristics on consumers’ preferences was evaluated, in each of the three countries, through three seemingly unrelated (SUR) regression models. Each model applied as dependent variables the differences between consumers’ WTP for FRG and conventional wines, in the informal and formal purchase occasions.

## 3. Materials and methods

### 3.1 Data collection and analysis

**Fig 1:**
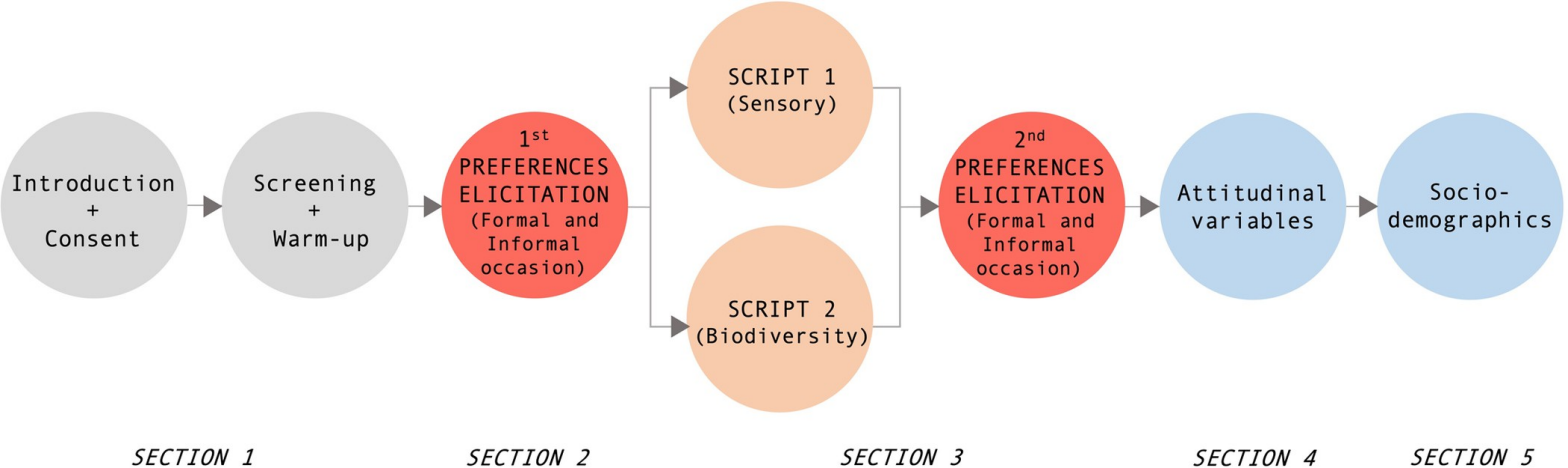
Survey structure and procedure.

**Fig 2:**
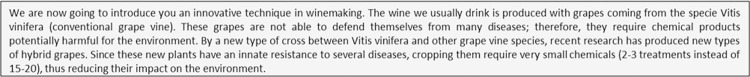
Basic information script.

In May 2021, an online experimental survey was completed by 2,466 regular wine consumers in three countries, namely, Italy (*n* = 752), the UK (*n* = 858), and the USA (*n* = 856). The survey was distributed through a professional market research company through their private, online panel. Country-specific sampling quotas were set based on age, gender, and geographic area of residence (*i*.*e*., region or state, depending on the specific country). Respondents who purchased wine less than once in the last four months and drank wine less than once in the last month were screened out. The study received a formal ethical waiver from the University of Padova in March 2021, and the research fully followed the principles stated by the Declaration of Helsinki. A pretest survey provided information on the respondents’ ability to understand the WTP elicitation format and the two information scripts. Some adjustments were made before running the final survey. The final questionnaire (S1 File) was organized in five sections (Fig 1). Following a brief introduction, the first section included screening and warm-up questions about participants’ wine consumption and purchasing frequency. The second section collected information on individuals’ willingness to pay (WTP) for both conventional wine and FRG wine. WTP was elicited two times, for an informal occasion at home (*e*.*g*., dinner with family or friends) and a formal occasion (*e*.*g*., business dinner with colleagues or a gift). To this end, a specific procedure was applied based on a payment ladder (specifically a click-and-drag slider, also considering cents). First, individuals were asked to state the amount of money they spent on the last 0.75 L bottle of wine for an informal occasion and a formal occasion. For each country sample, the price was expressed in the national currency (€ for Italy, £ for the UK, $ for the USA). It is also worth noting that no mention was made of other wine characteristics (such as origin, color, grape variety, and vintage). Subsequently, a brief information script (hereafter basic information) was provided regarding FRG wines (Fig 2). Then, participants were asked to state the maximum amount they were willing to pay for a 0.75 L bottle of FRG wine by using the same click-and-drag slider previously applied to conventional wine. To mitigate hypothetical bias, before answering the payment scale question, participants were asked to imagine that they were in the store where they generally purchase wine and were provided with a cheap talk script [37]. The WTP distributions were analyzed via parametric and non-parametric tests (*i*.*e*., *t* test, Mann-Whitney test, and Kolmogorov-Smirnov test).

**Fig 3:**
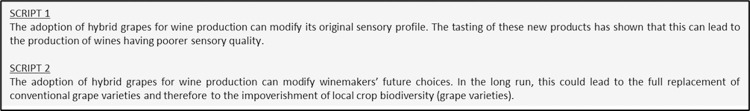
Additional information scripts.

In the third section of the questionnaire, after the presentation of two information scripts (Fig 3) on the potential drawbacks of FRG wines, a second collection of WTP for FRG wines was performed. More in detail, a between-subjects design was applied with two additional information scripts, each randomly assigned to half of the national samples. In particular, the first additional information script regarded the potential modification of FRG wine’s sensory profile (hereafter sensory information), whereas the second informed the consumers of the potential environmental impact of FRG wines, namely, the reduction in the local crop biodiversity due to the replacement of conventional grape varieties with fungus-resistant vines (hereafter biodiversity information). Subsequently, participants were asked to imagine that they were in the store where they normally purchase wine and to state again the maximum amount that they were willing to pay for a 0.75 L bottle of FRG wine, with the same click-and-drag scale previously used. Considering the double-censored structure of the dependent variables (WTP for formal and informal FRG wines) and its panel nature (same individual providing multiple evaluations) a random effects tobit model was estimated [38]. Specifically, the random effects tobit model equation was:

WTPij=Xijβ+ui+εij (1)

where WTP_*ij*_ is participant *i*’s left and right censored WTP for FRG wine *j*; X*ij* represents the vector of independent variables (formal and informal occasion) and information treatment; *β* is the estimated parameters; *u*_*i*_ is the unobserved random individual effect which varies across each individual *i* but not FRG wine *j*, thus capturing the correlation between bids submitted by the same individual; and *ε*_*ij*_ is the random error term.

The fourth section included three attitudinal scales, namely, wine involvement (WI), abbreviated food technology neophobia (AFTN), and sustainability concern (SC), collected through 7-point anchored items. Eventually, the last section included sociodemographic questions, namely, age, sex, household income, household size, geographic area of residence, education, occupation, whether they lived in a wine area, and their most frequent wine purchasing and consumption location.

The WI scale was adapted from Mittal and Lee [39] and was composed of six 7-point items (1 = strongly disagree; 7 = strongly agree), *e*.*g*., “I have a strong interest in wine”. The AFTN scale was derived from Schnettler et al. [40] and was composed of nine 7-point items (1 = strongly disagree; 7 = strongly agree), *e*.*g*., “New foods are not healthier than traditional foods”; after data collection, the fifth item was reversed. Finally, the SC scale by Grunert et al. [41] was used, which was composed of fourteen 7-point items (1 = only slightly concerned; 7 = extremely concerned), to measure participants’ level of concern toward both social and environmental sustainability issues. Examples include “The use of child labor in food production” and “Deforestation of the rainforest”. The final scales were derived by averaging the items into a composite score (Table 1). Cronbach’s α revealed a strong internal consistency for the three scales (higher than 0.8 for all scales). To capture the effect of individual characteristics on acceptance in each country, three SUR models were used to assess the impact of psychometric and sociodemographic characteristics on the differences between WTP of FRG (basic information only) and the price paid for the last purchased conventional wine bottle in the informal and formal consumption occasions (in the three countries samples). SUR is a multivariate linear regression model particularly useful in contexts where the estimation of a system of equations is required [42] due to the independence of the error terms across individuals and their correlation across equations. Similar approaches for CV food-data have been applied by Baldi et al. [43], Bir et al. [44] and Van Doorn and Verhoef [45]. In the Eq (2), *i* represents the *i-th* respondent, *x* is a vector of explanatory variables, and *e* is the error term, assumed to be independent across individuals and correlated across equations. For parsimony purposes, the selected models include only statistically significant coefficients. Statistical and graphical analyses were performed using STATA v.15.


$$\{{\Delta WTP}_{INFORMAL,i}\;{\Delta WTP}_{FORMAL,i}=x^{\prime }\beta_{INFORMAL}+e_{INFORMAL,i}=x^{\prime }\beta_{FORMAL}+e_{FORMAL,i}$$ (2)

**Table 1:** Attitudinal scale statistics (mean, standard deviation, and Cronbach’s α).

	ITALY			UK			USA		
	M	S.D.	α	M	S.D.	α	M	S.D.	α
**Wine Involvement (WI)**	5.23	1.25	0.95	5.03	1.20	0.92	5.09	1.28	0.92
**Abbreviated Food Technology Neophobia (AFTN)**	4.69	0.99	0.84	4.46	0.91	0.80	4.48	1.06	0.83
**Sustainability Concern (SC)**	5.70	1.02	0.95	5.44	1.09	0.94	5.25	1.18	0.93

Note: Scale anchoring for WI and AFTN: 1 = strongly disagree to 7 = strongly agree. Anchoring for SC: 1 = only slightly concerned to 7 = extremely concerned.

### 3.2 Sample characteristics

Table 2 reports the core characteristics of the respondents; additional information is presented in S1 Table. All samples were equally divided into males and females. Approximately half of the sample hold a household income level in the range of 2,000–4,000 € in Italy, 2,000–4,000 £ in the UK, and 3,700–7,400 $ in the USA, also reflecting a medium economic class level. Compared to Italy, where high school is the upper educational level, the majority of people in the UK and USA hold a university degree (37% and 49%, respectively). In each sample, the mean number of family members is 2.9 for Italy and the UK and 2.8 for the USA, and participants are mostly employed. In Italy, more than half of the sample claimed to live in a wine region, in contrast to both the UK and the USA. In addition, the participants indicated a high wine buying frequency: approximately 50% of the respondents in Italy and the UK declared they bought wine at least once a week, while in the USA, 56% of respondents stated that they bought wine at least 2–3 times per month. Regarding wine consumption frequency, in Italy, 91% of respondents drink wine at least once a week (and 35% every day), while in the UK and USA, this percentage is lower (80% and 71%, respectively). In every sample, individuals mainly buy wine at the supermarket or hypermarket or discount, followed by wineries (Italy and the UK) and wine bars (the USA and UK). Finally, approximately 80% of respondents in each sample drink wine mainly at home, followed by friends/relatives’ homes.

**Table 2:** Sociodemographic characteristics of Italian, UK, and US samples.

		Italy (%)	UK (%)	USA (%)
**Sex**	male	49.3	49.0	48.9
	female	50.7	51.0	50.7
	not revealed	-	-	0.4
**Age**	18–24 years (21–24 in USA)	9.4	13.2	7.2
	25–34 years	14.8	18.8	20.4
	35–44 years	18.4	18.1	18.9
	45–54 years	22.1	19.3	17.8
	55–64 years	18.8	16.7	18.9
	65–75 years	16.5	13.9	16.8
**Household income**	<2000 € (<2000 £; <3700 $)	45.5	33.1	38.6
	2000–4000 € (2000–4000 £; 3700–7400 $)	45.9	46.7	45.0
	>4000 € (>4000 £; >7400 $)	8.6	20.2	16.4
**Education**	primary school	0.3	1.0	1.6
	secondary school	9.6	19.1	2.9
	high school	53.2	27.0	31.3
	university	30.6	37.2	49.4
	post-graduate education	6.3	15.7	14.8
**Occupation**	employee	46.1	58.3	43.5
	free lance	3.1	2.0	1.7
	student	4.7	3.0	1.9
	housewife	11.0	5.8	10.4
	retired	12.9	13.6	22.0
	unemployed	10.1	4.1	8.2
	self employed	6.1	6.6	6.4
	business owner	2.5	5.6	4.7
	other	3.5	1.0	1.2
**Wine buying frequency**	once every 4 months	1.6	2.8	6.3
	once every 2–3 months	8.0	8.0	15.7
	once a month	14.6	16.7	22.0
	2–3 times/month	25.8	22.6	25.0
	once a week	33.4	32.4	16.7
	2–3 times/week	16.6	17.5	14.3
**Wine consumption frequency**	once a month	2.7	6.1	10.9
	2–3 times/month	6.0	14.5	18.6
	once a week	12.1	22.6	18.1
	2–3 times/week	26.3	32.2	26.6
	4–5 times/week	18.4	15.1	13.8
	everyday	34.5	9.5	12.0

## 4. Results

### 4.1. Consumers’ acceptance of FRG wines

**Fig 4:**
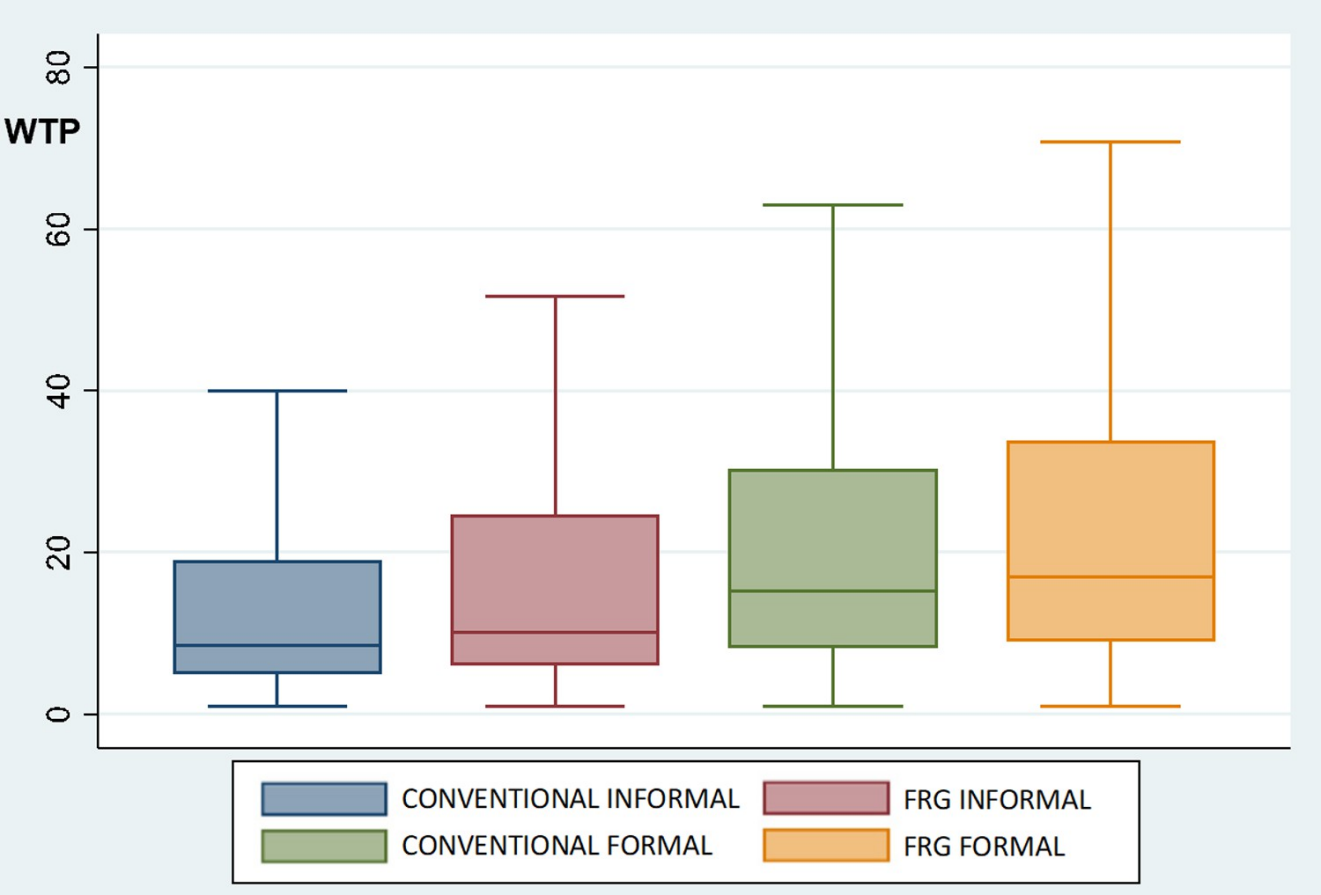
Boxplots of WTP distributions (€) for conventional and FRG wines in Italy (N = 752). Notes: CONVENTIONAL INFORMAL and CONVENTIONAL FORMAL = price paid (€) for the last conventional wine purchased for an informal and a formal occasion, respectively. FRG INFORMAL and FRG FORMAL = WTP (€) for FRG wine for informal and formal occasions, respectively, as expressed before treatment. According to *t* test, Mann-Whitney test, and Kolmogorov-Smirnov test, there is a statistically significant difference (*p*<0.05) between CONVENTIONAL INFORMAL and FRG INFORMAL, while CONVENTIONAL FORMAL and FRG FORMAL are not statistically different.

**Fig 5:**
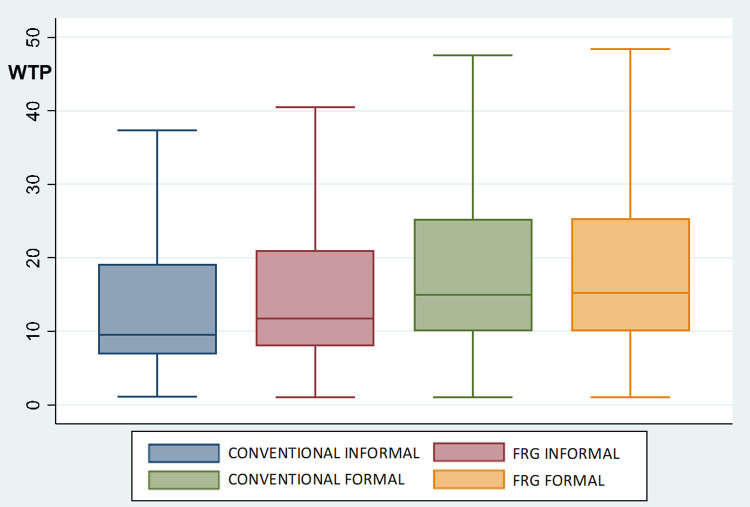
Boxplots of WTP distributions (*£*) for conventional and FRG wines for the UK (N = 858). Notes: CONVENTIONAL INFORMAL and CONVENTIONAL FORMAL = price paid (£) for the last conventional wine purchased for an informal and a formal occasion, respectively. FRG INFORMAL and FRG FORMAL = WTP (£) for FRG wine for informal and formal occasions, respectively, as expressed before treatment. According to *t* test, Mann-Whitney test, and Kolmogorov-Smirnov test, there is a statistically significant difference (*p*<0.05) between CONVENTIONAL INFORMAL and FRG INFORMAL, while CONVENTIONAL FORMAL and FRG FORMAL are not statistically different.

**Fig 6:**
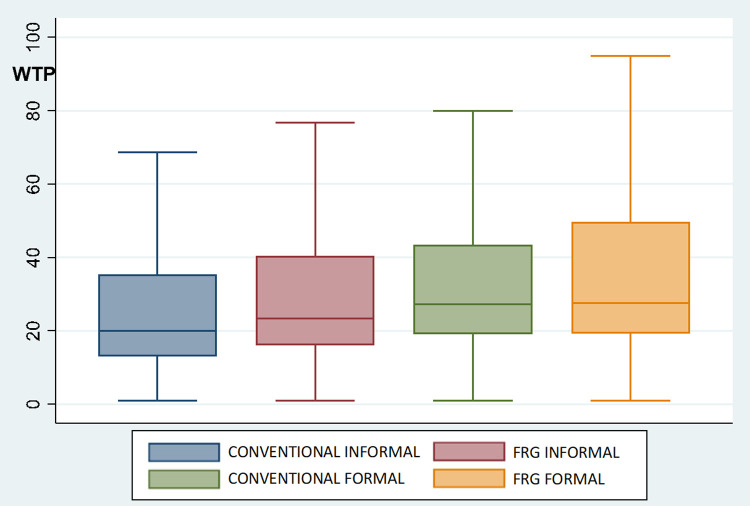
Boxplots of WTP distributions (*$*) for conventional and FRG wines for the USA (N = 856). Notes: CONVENTIONAL INFORMAL and CONVENTIONAL FORMAL = price paid ($) for the last conventional wine purchased for an informal and a formal occasion, respectively. FRG INFORMAL and FRG FORMAL = WTP ($) for FRG wine for informal and formal occasions, respectively, as expressed before treatment. According to *t* test, Mann-Whitney test, and Kolmogorov-Smirnov test, there is a statistically significant difference (*p*<0.05) between CONVENTIONAL INFORMAL and FRG INFORMAL, while CONVENTIONAL FORMAL and FRG FORMAL are not statistically different.

To measure consumers’ acceptance of FRG wines in Italy, the UK and USA and explore whether informal or formal consumption occasions determine differences in consumers’ preferences (RQ1 and RQ2), the WTP distributions were analyzed via parametric and nonparametric tests. Figs 4–6 depict the distribution of respondents’ WTP for conventional and FRG wines on informal and formal occasions in the three countries. The WTP distributions reveal a similar pattern in Italy, the UK and USA, which shows the general acceptance of FRG wines among consumers. On formal occasions, the average WTP for FRG wine and conventional wine is not significantly different. In particular, on formal occasions, in Italy, the mean WTP for conventional wine is 23.9€ (S.D. 22.3), while for FRG wine it is 24.5€ (S.D. 21.1); in the UK, the average WTP for conventional wine is 21.8£ (S.D. 19.0), while for FRG wine it is 22.1£ (S.D. 18.6); and in the USA, the mean WTP for conventional wine is 34.7$ (S.D. 23.7), while for FRG wine it is 35.3$ (S.D. 23.4). Considering the distributions of consumers’ WTP on informal consumption occasions, the outcomes show a higher WTP for FRG wine than for conventional wine. In particular, on informal occasions, in Italy, the average WTP for conventional wine is 18.1€ (S.D. 22.2), while for FRG wine it is 20.4€ (S.D. 22.0); in the UK, the average WTP for conventional wine is 16.9£ (S.D. 17.4), while for FRG wine it is 19.2£ (S.D. 18.5); and in the USA, the average WTP for conventional wine is 27.4$ (S.D. 21.6), while for FRG wine it is 31.8$ (S.D. 22.6). Differences between consumers’ WTP for FRG wines on informal and formal occasions are all statistically significant (according to *t* test, Mann-Whitney test, and Kolmogorov-Smirnov test), with consumers willing to pay more on formal consumption occasions than on informal consumption occasions.

### 4.2 Information effect and preference drivers

To assess the effects of different information on consumers’ preferences for FRG wines (RQ3), a random effect tobit regression was applied. More specifically, the tobit aimed to assess, in the full sample (*i*.*e*., three countries together) and both for the formal and informal consumption occasions, whether there is a difference in the effect on individuals’ WTP between information regarding the potential modification of FRG wine’s sensory profile, and information concerning the potential reduction in local crop biodiversity due to the replacement of conventional grape varieties with FRG vines.

Table 3 shows that different information scripts have diverse effects on the acceptance of FRG wine. Specifically, individuals who received sensory information had lower WTP than those who read the script with biodiversity information. The same relation occurred for both informal and formal consumption occasions.

**Table 3:** Influence of information scripts on FRG preferences (random effects tobit regression).

	WTP
**Sensory script**	-1.842***
**FRG_INF_**	-2.340***
***Constant***	26.469***
***Rho***	*0*.*847*
***Log likelihood***	*- 20288*.*274*
***Number of Obs***.	*4932*

Notes: Dependent variable is WTP for FRG wines after reading the information script; Asterisks represent statistical significance at the following levels

** *p*≤0.05 and

*** *p*≤0.01. Likelihood-ratio test of sigma u = 0: 3076.07***.

Three SUR models were used to assess, for each country and for both formal and informal occasions, individual characteristics affecting consumers’ acceptance of FRG wines (RQ4). We reiterate here that the linear payment ladder may have overestimated participants’ WTP; however, we draw on the findings of Lusk and Schroeder [46], whose study revealed that marginal WTP for an attribute change—obtained by taking the difference of WTP for two similar products—is not significantly different in hypothetical and non-hypothetical settings. Table 4 reveals the influence of certain sociodemographic variables and attitudinal scales on Italian, British, and American consumers’ preferences on informal and formal occasions. In Italy, the preference for FRG wines is positively influenced by individuals’ wine involvement (only on informal occasions) and sustainability concerns (only on formal occasions), while they are negatively influenced by their food technology neophobia. Considering the UK, on informal occasions, preferences are positively influenced by wine involvement and negatively influenced by consumers’ age, whereas on formal occasions, preference is positively influenced by individuals’ sustainability concerns and negatively influenced by a high score on the food technology neophobia scale. Last, in the USA, sustainability concerns and household income (the latter only for FRG consumed in informal occasions) increase consumers’ monetary preferences; instead, wine consumption frequency and being male (the latter only on formal occasions) decrease WTP.

**Table 4:** Influence of psychographic and socio-demographic characteristics on FRG preferences (seemingly unrelated regression coefficients).

	Italy				UK				USA			
	ΔWTP_INF_		ΔWTP_FOR_		ΔWTP_INF_		ΔWTP_FOR_		ΔWTP_INF_		ΔWTP_FOR_	
**WI**	1.003	***			0.673	***						
**AFTN**	-0.658	**	-1.545	***			-0.732	**				
**SC**			1.358	***			0.680	***	0.751	***	0.928	***
**Wine. Freq.**									-0.559	*	-0.950	***
**Age**					-0.319	*						
**Male**											-2.087	**
**Household income**									1.320	**		
***Equations***	*Obs*		*Parms*		*RMSE* ^*^*^							
**ΔWTP_INF_ (Italy)**	752		2		12.097							
**ΔWTP_FOR_ (Italy)**	752		2		13.083							
**ΔWTP_INF_ (UK)**	858		2		9.267							
**ΔWTP_FOR_ (UK)**	858		2		11.623							
**ΔWTP_INF_ (USA)**	856		3		13.008							
**ΔWTP_FOR_ (USA)**	856		3		16.624							

Notes: Dependent variables (for Italy, the UK and USA): ΔWTP_INF_ = WTP FRG–PRICE PAID FOR LAST PURCHASED CONVENTIONAL (before the information script, for an informal occasion); ΔWTP_FOR_ = WTP FRG–PRICE PAID FOR LAST PURCHASED CONVENTIONAL (before the information script, for a formal occasion). ^^:^ Root mean square error. Asterisks represent statistical significance at the following levels

* *p*≤0.1

** p≤0.05

*** *p*≤0.01. Breusch–Pagan test of independence: Chi-square 44.615*** (Italy); Chi-square 46.297*** (UK); Chi-square 84.928*** (USA).

## 5. Discussion

Although market data and scholarly insights [1] highlight the increased relevance of sustainable wine characteristics among consumers, individuals’ acceptance of novel production methods in this sector may be controversial [27]. To make the most of the disruptive potential of FRG wines to reduce pesticide use, understanding the elements conditioning consumers’ perception of this innovation is crucial for the current, early stage of commercialization.

The present study analyzed consumers’ acceptance of FRGs by surveying large samples of Italian, British and American regular wine drinkers and comparing individuals’ preferences for conventional wines with preferences for FRG wines. The research also explored whether acceptance is influenced by the consumption occasion (informal or formal), by receiving different types of information about the product, and by individual attitudinal characteristics. The findings consolidate previous exploratory insights [25–27], showing that consumers are willing to pay the same amount for FRG wines as they are for their conventional counterparts—or even more—in an informal situation. This outcome holds true irrespective of the country, rejecting the hypothesis of geography-dependent differences in overall FRG acceptance.

The findings confirm that regular wine drinkers behave differently depending on the specific circumstance for which they are buying a product [30]. In the case of FRG wine, there stands to be a marked difference between informal and formal occasions, with individuals less prone to pay a price premium in the latter situation. This tendency is in line with other studies describing purchasing a wine for a gift or drinking outside the domestic context as a critical moment [31,32], for which wine quality becomes of utmost importance. A potential inference is that consumers may associate with FRG wines the risk of making a negative impression in a social situation, for instance, due to their unknown sensory profile. Accordingly, the findings also suggest that informing individuals about the potential effects of FRG on the sensory characteristics of wine may have a negative effect on their WTP, more so than alternative negative information related to agricultural biodiversity concerns. This outcome holds true both for informal and formal situations, confirming the evidence that wine taste is always important, irrespective of the wine drinking situation [47]. In addition, this finding confirms FRG variety breeders’ concerns about the paramount relevance of maintaining the organoleptic and sensorial properties of traditional wines to preserve their acceptance among consumers [14].

The analysis of respondents’ WTP for FRG wines showed several individual characteristics effectively driving preferences. Sustainability concerns play a key role in fostering support for FRG wines, confirming the significance of this feature in the modern wine market [1]. Wine involvement also had a positive effect, with individuals for which wine is highly relevant being more prone to pay a premium price for FRG wines. However, this evidence is limited to informal occasions, suggesting personal curiosity for these new wines as a possible WTP booster. The outcomes also show that aversion to novel food technologies may lead to a lower acceptance of FRG wines, confirming this factor as one of the main barriers to their uptake in the wine market [20].

The current study has several important limitations. First, measuring individuals’ stated preferences (WTP collected through an online survey) is strongly subject to hypothetical bias [48] and social desirability bias [49]. Additionally, the linear payment ladder is particularly keen to issues of range and centering bias [33]. Furthermore, the investigated samples purposely included only regular wine consumers participating to a marketing company panel and thus provide a partial picture of the wider market. Insights should also be gathered on occasional wine consumers, whose views could be substantially diverse. Finally, the study, due to its design, underestimates other important drivers of consumer preferences (*e*.*g*., origin, brand, wine sensory characteristics).

## 6. Conclusions

The modern wine industry has been increasingly urged by policy-makers, large retailers and consumers to improve its sustainability performance; nevertheless, significant results in terms of both lower environmental impacts and lower social impacts remain difficult to achieve in wine production.

In recent years, scientific research has led to the development of new wines produced with fungus-resistant grape varieties, which can be obtained with traditional genetic techniques, thus avoiding objections about the use of genetic transformation technologies. FRG wines represent radical innovations in a sector and market still strongly dominated by tradition, grape varieties, and place-specific origin. Indeed, currently, both the areas planted with FRG varieties and the experience in FRG winemaking are limited. However, disclosing insights into consumer acceptance of these wines and the core preference drivers might foster winery adoption and policy-makers’ active engagement, providing effective guidelines for practical information campaigns and highlighting the most promising market targets.

Further studies should delve into consumer acceptance of FRG wines, extending the scope to additional market segments and including relevant attributes (for example brand, origin, and sensory descriptors) in the preference elicitation procedure. Moreover, future research should aim to overcome the key shortcomings of this study, as self-selection bias and WTP overestimation.

FRG wines represent a sustainable innovation that has important potentialities. For instance, fungus-resistant grape varieties can represent a possible mitigation strategy against climate change: indeed, the lower number of treatments required and therefore the lower use of pesticides reflect both a reduced use of potential pollutants in viticulture and a reduced carbon footprint. Nevertheless, it is uncertain whether their diffusion and success will be geographically homogeneous in the future, and it is possible to suppose that the greatest interest towards this innovation will occur in areas where the weather and climate conditions normally require a greater use of pesticides in viticulture.

## Supporting information

S1 File Questionnaire. (DOCX)

S1 Table Additional sample characteristics. (DOCX)
