# Supplementary material for: Consumer acceptance of fungus-resistant grape wines: Evidence from Italy, the UK, and the USA
Source: PLoS One. 2022 Apr 27;17(4):e0267198. doi: 10.1371/journal.pone.0267198 (PMC9045640; doi:10.1371/journal.pone.0267198)
Supplement: S1 File — (DOCX) [file pone.0267198.s001.docx]

**S1 File. Questionnaire**

Thank you for participating in this survey on wine consumption carried out by the University of Padua and the University of Naples Federico II.

This survey has no commercial purposes. The questionnaire is anonymous, information will be analysed in an aggregated way and results will be used only for scientific purposes.

Filling in the questionnaire will require about 16 minutes. There are no right or wrong answers, we are only interested in your personal opinion.

**Section 1**

Please, fill in the questionnaire only if you are more than 18 years old and drink wine (21 years if USA).

Do you agree to participate?

|  | I agree |
| --- | --- |
|  | I do not agree |

Sex

|  | male |
| --- | --- |
|  | female |
|  | other |

You live in… [list of regions/state] (drop down list)

Please, respond to a couple of questions concerning your consumption and purchase habits related to wine.

How frequently do you buy wine?

|  | Everyday |
| --- | --- |
|  | 4-5 times a week |
|  | 2-3 times a week |
|  | Once a week |
|  | 2-3 times a month |
|  | Once a month |
|  | More than once a year |
|  | Never |

How frequently do you drink wine?

|  | Everyday |
| --- | --- |
|  | 4-5 times a week |
|  | 2-3 times a week |
|  | Once a week |
|  | 2-3 times a month |
|  | Once a month |
|  | More than once a year |
|  | Never |

**Section 2**

Think about the last bottle of wine (0.75 L) you purchased to drink for an informal occasion at your home with family or friends, what was the price you paid for that bottle?

Move the cursor of the slider to indicate the value in euro (or £ if UK; or $ if USA) and the second cursor to specify the cents.

SLIDER 1-100, step 1 € (or £ if UK; or $ if USA)

SLIDER 0-99, step 1 cent

Think about the last bottle of wine (0.75 L) you purchased to drink for a formal occasion (business, colleagues) or a gift, what was the price paid for that bottle?

Move the cursor of the slider to indicate the value in euro (or £ if UK; or $ if USA) and the second cursor to specify the cents.

SLIDER 1-100, step 1 € (or £ if UK; or $ if USA)

SLIDER 0-99, step 1 cent

*We are now going to introduce you to an innovative technique in winemaking.*

*The wine we usually drink is produced with grapes coming from the species Vitis vinifera (conventional grape vine). These grapes are not able to defend themselves from many diseases; therefore, they require chemical products potentially harmful for the environment. By a new type of cross between Vitis vinifera and other grape vine species, recent research has produced new types of hybrid grapes. Since these new plants have an innate resistance to several diseases, cropping them requires very small chemicals (2-3 treatments instead of 15-20), thus reducing their impact on the environment.*

Now we would like you to imagine that you are in the store where you normally buy wine for an informal occasion at your home (e.g., a dinner at home with family or friends). What is your maximum willingness to pay for a 0.75 L wine bottle produced with hybrid grapes?

According to recent studies, sometimes individuals give some responses, but then they behave differently in real life. Please, when declaring your willingness to pay, respond exactly as if you were in a real shop.

Move the cursor of the slider to indicate the value in euro (or £ if UK; or $ if USA) and the second cursor to specify the cents.

SLIDER 1-100, step 1 € (or £ if UK; or $ if USA)

SLIDER 0-99, step 1 cent

Now we would like you to imagine that you are in the store where you normally buy wine for a formal occasion (e.g., a business dinner with colleagues) or a gift. What is your maximum willingness to pay for a 0.75 L wine bottle produced with hybrid grapes?

Move the cursor of the slider to indicate the value in euro (or £ if UK; or $ if USA) and the second cursor to specify the cents.

SLIDER 1-100, step 1 € (or £ if UK; or $ if USA)

SLIDER 0-99, step 1 cent

**Section 3**

Now, we would like to give you more information on hybrid grapes. Please, read carefully the text below

*SCRIPT 1*

*The adoption of hybrid grapes for wine production can modify its original sensory profile. The tasting of these new products has shown that this can lead to the production of wines having poorer sensory quality.*

*SCRIPT 2*

*The adoption of hybrid grapes for wine production can modify winemakers’ future choices. In the long run, this could lead to the full replacement of conventional grape varieties and therefore to the impoverishment of local crop biodiversity (grape varieties).*

Once again, we would like you to imagine that you are in the store where you normally buy wine for an informal occasion at your home (e.g., dinner at home with family or friends). What is your maximum willingness to pay for a 0.75 L wine bottle produced with hybrid grapes?

Previously you said you would pay XX € (or £ if UK; or $ if USA), what is now your maximum willingness to pay?

Move the cursor of the slider to indicate the value in euro (or £ if UK; or $ if USA) and the second cursor to specify the cents.

SLIDER 1-100, step 1 € (or £ if UK; or $ if USA)

SLIDER 0-99, step 1 cent

Once again, we would like you to imagine that you are in the store where you normally buy wine for a formal occasion (e.g., a business dinner with colleagues) or a gift. What is your maximum willingness to pay for a 0.75 L wine bottle produced with hybrid grapes?

Previously you said you would pay XX (or £ if UK; or $ if USA), what is now your maximum willingness to pay?

Move the cursor of the slider to indicate the value in euro (or £ if UK; or $ if USA) and the second cursor to specify the cents.

SLIDER 1-100, step 1 € (or £ if UK; or $ if USA)

SLIDER 0-99, step 1 cent

**Section 4**

Respond now to some questions on your general wine interest.

Please, indicate your level of agreement with the following opinions, using a scale from 1 to 7 (1 = strongly disagree; 7 = strongly agree).

|  | 1 | 2 | 3 | 4 | 5 | 6 | 7 |
| --- | --- | --- | --- | --- | --- | --- | --- |
| I have a strong interest in wine |  |  |  |  |  |  |  |
| Wine is very important to me |  |  |  |  |  |  |  |
| For me, wine do matter |  |  |  |  |  |  |  |
| I would choose my wine very carefully |  |  |  |  |  |  |  |
| Deciding which wine to buy would be an important decision for me |  |  |  |  |  |  |  |
| Which wine I buy matters to me a lot |  |  |  |  |  |  |  |

Please, indicate your level of agreement with the following opinions, using a scale from 1 to 7 (1 = strongly disagree; 7 = strongly agree).

|  | 1 | 2 | 3 | 4 | 5 | 6 | 7 |
| --- | --- | --- | --- | --- | --- | --- | --- |
| New foods are not healthier than traditional foods |  |  |  |  |  |  |  |
| The benefits of new food technologies are often grossly overstated |  |  |  |  |  |  |  |
| There are plenty of tasty foods around so we do not need to use new food technologies to produce more |  |  |  |  |  |  |  |
| New food technologies decrease the natural quality of food |  |  |  |  |  |  |  |
| New food technologies are unlikely to have long term negative health effects |  |  |  |  |  |  |  |
| New food technologies may have long term negative environmental effects |  |  |  |  |  |  |  |
| It can be risky to switch to new food technologies too quickly |  |  |  |  |  |  |  |
| Society should not depend heavily on technologies to solve its food problems |  |  |  |  |  |  |  |
| There is no sense trying out high-tech food products because the ones I eat are already good enough |  |  |  |  |  |  |  |

Please, indicate your level of concern for the following issues, using a scale from 1 to 7 (1 = only slightly concerned; 7 = extremely concerned).

|  | 1 | 2 | 3 | 4 | 5 | 6 | 7 |
| --- | --- | --- | --- | --- | --- | --- | --- |
| The use of child labour in food production |  |  |  |  |  |  |  |
| Deforestation of the rainforest |  |  |  |  |  |  |  |
| Starvation and malnutrition in the world population |  |  |  |  |  |  |  |
| The use of pesticides used in food production |  |  |  |  |  |  |  |
| Poor treatment of animals in food production |  |  |  |  |  |  |  |
| Environmental damage caused by human use of land and water |  |  |  |  |  |  |  |
| The amount of food that is wasted |  |  |  |  |  |  |  |
| Using too much of the world’s natural resources for food production |  |  |  |  |  |  |  |
| Poor working conditions and wages for food producers |  |  |  |  |  |  |  |
| Packaging that is not recyclable |  |  |  |  |  |  |  |
| The amount of packaging used on products |  |  |  |  |  |  |  |
| Carbon emissions caused by food production |  |  |  |  |  |  |  |
| The amount of energy used when transporting food products |  |  |  |  |  |  |  |
| The amount of energy used when cooking food products |  |  |  |  |  |  |  |

**Section 5**

Please, respond to some questions concerning your purchase habits related to wine.

How old are you? [ITA: 18-75; UK: 18-75; USA: 21-75]

Number of family members (including you) ________ (open-ended)

Household income (gross monthly income)

|  | <2000 € (<2000 £; <3700 $) |
| --- | --- |
|  | 2000-4000 € (2000-4000 £; 3700-7400 $) |
|  | >4000 € (>4000 £; >7400 $) |

Education level

|  | primary school |
| --- | --- |
|  | secondary school |
|  | high school |
|  | University |
|  | post-graduate education |

Occupation

|  | business owner |
| --- | --- |
|  | self employed |
|  | employee |
|  | freelance |
|  | student |
|  | housewife |
|  | retired |
|  | unemployed |
|  | other |

Do you live in a wine production area?

|  | yes |
| --- | --- |
|  | no |

Where do you purchase wine more frequently?

|  | supermarket, hypermarket, discount |
| --- | --- |
|  | from the producer (winery) |
|  | wine bar |
|  | online |

Where do you drink wine most frequently?

|  | home |
| --- | --- |
|  | friends/relatives’ house |
|  | restaurant |
|  | wine bar |
