# Supplementary material for: Consumer acceptance of fungus-resistant grape wines: Evidence from Italy, the UK, and the USA
Source: PLoS One. 2022 Apr 27;17(4):e0267198. doi: 10.1371/journal.pone.0267198 (PMC9045640; doi:10.1371/journal.pone.0267198)
Supplement: S1 Table — (DOCX) [file pone.0267198.s002.docx]

**S2 Table. Additional sample characteristics.**

|  | | Italy (%) | | UK (%) | USA (%) |
| --- | --- | --- | --- | --- | --- |
| Living in a wine area | no | | 46.4 | 89.2 | 77.6 |
|  | yes | | 53.6 | 10.8 | 22.4 |
| Geographic area of residence | South | | 31.7 |  |  |
|  | Centre | | 22.6 |  |  |
|  | North-West | | 26.6 |  |  |
|  | North-East | | 19.1 |  |  |
|  | East Midlands | |  | 6.3 |  |
|  | East of England | |  | 8.2 |  |
|  | Greater London | |  | 13.2 |  |
|  | North-East | |  | 5.2 |  |
|  | North-West | |  | 11.0 |  |
|  | Northern Ireland | |  | 2.7 |  |
|  | Scotland | |  | 8.2 |  |
|  | South-East | |  | 14.5 |  |
|  | South-West | |  | 8.0 |  |
|  | Wales | |  | 5.1 |  |
|  | West Midlands | |  | 10.8 |  |
|  | Yorkshire and the Humber | |  | 6.8 |  |
|  | Connecticut, Maine, Massachusetts, New Hampshire, Rhode Island, Vermont | |  |  | 5.2 |
|  | New Jersey, New York, Puerto Rico, US Virgin Islands | |  |  | 11.1 |
|  | Delaware, District of Columbia, Maryland, Pennsylvania, Virginia, West Virginia | |  |  | 10.5 |
|  | Alabama, Florida, Georgia, Kentucky, Mississippi, North Carolina, South Carolina, Tennessee | |  |  | 20.4 |
|  | Illinois, Indiana, Michigan, Minnesota, Ohio, Wisconsin | |  |  | 16.9 |
|  | Arkansas, Louisiana, New Mexico, Oklahoma, Texas | |  |  | 12.0 |
|  | Iowa, Kansas, Missouri, Nebraska | |  |  | 4.0 |
|  | Colorado, Montana, North Dakota, South Dakota, Utah, Wyoming | |  |  | 2.8 |
|  | Arizona, California, Hawaii, Nevada, American Samoa, Guam, Northern Mariana Islands | |  |  | 14.1 |
|  | Alaska, Idaho, Oregon, Washington | |  |  | 3.0 |
| Most frequent wine purchasing location | supermarket, hypermarket, discount | | 58.8 | 76.0 | 50.7 |
|  | from the producer (winery) | | 19.0 | 8.3 | 7.5 |
|  | wine bar | | 17.8 | 8.2 | 34.9 |
|  | online | | 4.1 | 7.4 | 6.9 |
|  | other | | 0.3 | 0.1 | 0.0 |
| Most frequent wine consumption location | home | | 77.9 | 81.1 | 80.7 |
|  | friends/relatives’ home | | 12.4 | 8.9 | 10.2 |
|  | restaurant | | 7.5 | 7.3 | 7.8 |
|  | wine bar | | 2.1 | 2.3 | 1.2 |
|  | other | | 0.1 | 0.4 | 0.1 |
| Number of family members – mean (standard deviation) | | | 2.9 (1.2) | 2.9 (1.4) | 2.8 (1.7) |
